# Recent advances of the mammalian target of rapamycin signaling in mesenchymal stem cells

**DOI:** 10.3389/fgene.2022.970699

**Published:** 2022-08-30

**Authors:** Huarui Cai, Zhongze Wang, Wenhan Tang, Xiaoxue Ke, Erhu Zhao

**Affiliations:** ^1^ State Key Laboratory of Silkworm Genome Biology, College of Sericulture, Textile and Biomass Sciences, Southwest University, Chongqing, China; ^2^ Cancer Center, Medical Research Institute, Southwest University, Chongqing, China

**Keywords:** mTOR, mesenchymal stem cells, differentiation, immune response, ageing-related diseases, therapeutic target

## Abstract

Mammalian target of rapamycin (mTOR) is a serine/threonine kinase involved in a variety of cellular functions, such as cell proliferation, metabolism, autophagy, survival and cytoskeletal organization. Furthermore, mTOR is made up of three multisubunit complexes, mTOR complex 1, mTOR complex 2, and putative mTOR complex 3. In recent years, increasing evidence has suggested that mTOR plays important roles in the differentiation and immune responses of mesenchymal stem cells (MSCs). In addition, mTOR is a vital regulator of pivotal cellular and physiological functions, such as cell metabolism, survival and ageing, where it has emerged as a novel therapeutic target for ageing-related diseases. Therefore, the mTOR signaling may develop a large impact on the treatment of ageing-related diseases with MSCs. In this review, we discuss prospects for future research in this field.

## 1 Introduction

The mTOR protein plays an essential role in cell metabolism, promoting cell proliferation and survival through changes in energy and substance metabolism (Zhao et al., 2016; Karagianni et al., 2022). The mTOR protein is associated with the phosphoinositide 3-kinase (PI3K)/protein kinase B (Akt) signaling pathway, which is involved in hormone, growth factor and nutrient signal transduction (Holz et al., 2005; Murugan, 2019; Huang, 2020; Holz et al., 2021). Environmental signaling activates the mTOR pathway to regulate kinds of fundamental processes required for cell growth, metabolism, regeneration and ageing, among others (Murugan, 2019). mTOR is often dysregulated in human cancers, and somatic mutations that induce mTOR activation have recently been identified in several types of human cancers, suggesting that mTOR is a therapeutic target (Murugan, 2019; Huang, 2020; Zou et al., 2020). Over time, research on the mTOR signaling pathway has become a hot topic in various fields, such as metabolism, genomics, pharmacology and inhibitors. Studies have focused on not only animal models but also human-related diseases, such as cancers and neurodegenerative diseases. In recent years, the mTOR signaling pathway has been increasingly studied in stem cells, especially mesenchymal stem cells (MSCs).

The term “mesenchymal stem cell” was coined in the end of the 20th century, and the criteria for defining MSCs were issued by the International Society for Cellular Therapy in 2006 (Andrzejewska et al., 2019). This development was followed by numerous preclinical studies on the potential therapeutic properties of MSCs, such as immune regulation, nutritional support, the ability to spontaneously differentiate into connective tissue cells, and the ability to differentiate into most cell types under specific induction conditions (Andrzejewska et al., 2019). MSCs are widely used in regenerative medicine and oncology, in part because of the lack of conventional therapies for these demanding and expensive diseases. It has been speculated that MSCs are intermediate forms of subpopulations or pericytes, but there is still no convincing molecular evidence to confirm this hypothesis (Caplan, 2008; Blocki et al., 2013).

At present, human MSCs have been used in the clinical treatment of various diseases (Luzzani and Miriuka, 2017). In earlier studies, the efficacy of MSC therapy was primarily attributed to the ability of these cells to locally transplant and differentiate into multiple tissue types (Vizoso et al., 2017). However, with age, human brain function can also decline to cause certain senile neurodegenerative diseases, such as Alzheimer’s disease (AD) and Parkinson’s disease (PD) (Mezzaroba et al., 2019). The treatment, prevention and control of senile diseases are also major problems facing society. In this context, MSCs have been gradually used to prevent and treat senile diseases, and related studies have been performed. In this review, we summarize the latest advances in the rapidly evolving field of mTOR, discuss the composition of the mTOR signaling pathway and its related effects on MSCs, and provide a summary of the roles of the mTOR signaling pathway in MSC-mediated treatments of ageing-related diseases.

## 2 Composition and domain of mammalian target of rapamycin

**FIGURE 1:**
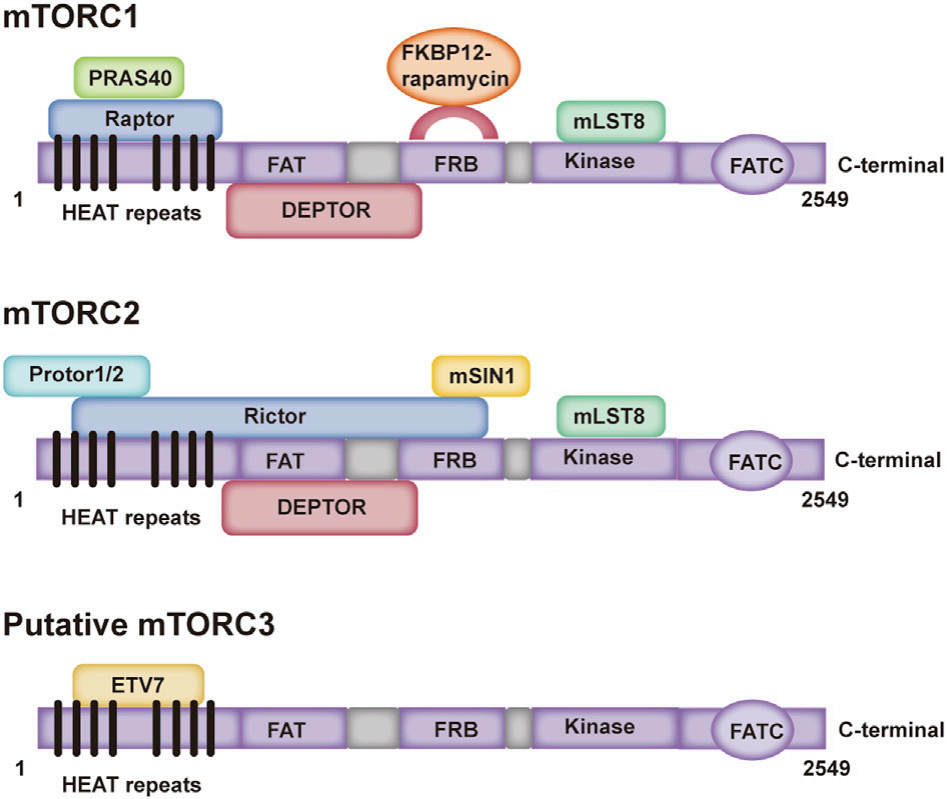
Domain structure of three mTOR complexes. PRAS40: A known Akt substrate is a 40 kDa proline-enriched protein; Raptor: Regulation related proteins of mTOR; FKBP-12: a prototype member of the immune affinity protein FKBP (FK506-binding protein) family capable of binding to the immunosuppressive drug FK506 (tacrolimus); mLST8: mammalian lethal with SEC13 protein eight; DEPTOR: it can interact with rictor; Protor: protein observed with rictor; Rictor: rapamycin-insensitive companion of mTOR; mSIN1: mammalian stress-activated protein kinase interacting protein; ETV7: ETS variant transcription factor 7.

The mTOR protein belongs to the PI3K-related kinase family and is encoded by the mTOR gene, which is an evolutionarily conserved serine/threonine kinase (Xiang et al., 2011). The mTOR protein has a carboxy terminal sequence with strong homology to the catalytic domain of PI3K, and acts as a protein kinase (Brunn et al., 1997). As a central signal aggregator, mTOR can transmit and integrate various signals, such as those from growth factors, nutrients, and cellular energy metabolism, and can balance anabolic and catabolic states in a negatively regulated manner (Feng et al., 2005; Shams et al., 2021). Mammals express one mTOR protein that serves as a core and essential component of three multisubunit complexes, namely, mTOR complex 1 (mTORC1), mTOR complex 2 (mTORC2) and a putative mTOR complex 3 (mTORC3) (Zou et al., 2020; Butt et al., 2019; el Hage and Dormond, 2021; Harwood et al., 2018; Liu and Sabatini, 2020). The mTORC1 complex is composed mainly of mTOR, Raptor, mLST8, DEPTOR and PRAS40, and mTORC2 is composed mainly of mTOR, Rictor, mLST8, DEPTOR and mSIN1 (Ding et al., 2013) (Figure 1). Moreover, previous studies have shown that the PNT domain of ETV7 binds with the mTOR domain to form putative mTORC3, which does not possess key components of mTORC1/2 (Raptor, Rictor, mSIN1 and mSLT8); but its size is similar to that of mTORC2 (Thoreen et al., 2009; Luo et al., 2015; Harwood et al., 2018; Chiarini et al., 2019; Takahara et al., 2020) (Figure 1). mTORC1, as a signal integrator, balances protein synthesis and degradation to regulate cell growth by sensing nutrients and growth factors, while mTORC2 is involved in the regulation of cell survival and cytoskeletal organization by acting through protein kinase B (Akt) (Loewith et al., 2002; Kim et al., 2017a; Rion et al., 2019; Ciolczyk-Wierzbicka et al., 2020; Liu and Sabatini, 2020; Popova and Jucker, 2021). Whereas rapamycin is a potent inhibitor of mTORC1, mTORC2 is resistant to rapamycin (Ding et al., 2013) [(Julien and Roux, 2010), (Lund-Ricard et al., 2020)]. Putative mTORC3 is also resistant to rapamycin, which can assemble on the basis of ETV7 expression in various cancers and increase tumor incidence and penetrance (Harwood et al., 2018).

## 3 Effects of mammalian target of rapamycin on mesenchymal stem cell differentiation

### 3.1 Mammalian target of rapamycin and adipogenesis

Lipid synthesis is a key nutrient metabolic pathway that allows organisms to remain active even when energy is limited (Caron et al., 2015; Wang et al., 2021). Adipogenic differentiation of MSCs is a key developmental process associated with metabolic homeostasis and nutritional signal transduction (Fernandez-Veledo et al., 2013; Lee et al., 2016). In adipose cells, mTOR plays a central role in protein synthesis and adipose tissue morphogenesis (Xiang et al., 2011). Activated by anabolic signals, the kinase mTOR plays a primary role in controlling lipid biosynthesis and metabolism in response to nutrition, and a key role in the formation of complexes that both promote fat formation and inhibit fat decomposition and oxidation, ultimately leading to the accumulation of triglycerides (Caron et al., 2015). Early reports have revealed that mTOR has a lipogenic effect, and can promote adipogenesis in white adipocytes, brown adipocytes and muscle satellite cells (Vila-Bedmar et al., 2010). mTOR activity is critical in the first stage of the differentiation of brown adipocytes, and adenosine monophosphate-activated protein kinase (AMPK)-mTOR crossover is a mediator of this process (Vila-Bedmar et al., 2010). Insulin activates mTORC1 through the PI3K/AKT pathway to regulate adipogenesis, and mTOR inhibition has a negative regulation of adipocyte differentiation and insulin signaling (Kim and Chen, 2004; Yu et al., 2008; Zhang et al., 2009; Xiang et al., 2011).

In recent years, studies have shown that mTOR complexes play important roles in increasing *de novo* adipogenesis in liver and adipose tissue. mTORC1 has a positively regulation of sterol regulatory element binding protein (SREBP). mTORC1 promotes SREBP expression, maturation, and nuclear localization through an S6K1-dependent pathway or phosphorylates the phospholipid acid phosphatase lipin-1 and controls its nuclear translocation (Porstmann et al., 2008; Duvel et al., 2010; Chakrabarti and Kandror, 2015). In addition, adipogenesis independent of mTORC1 has also been shown to be controlled by mTORC2, which phosphorylates AKT, which targets ATP-citrate lyase as a distinct substrate, thereby driving brown adipogenesis and *de novo* lipogenesis (Calejman et al., 2020).

**FIGURE 2:**
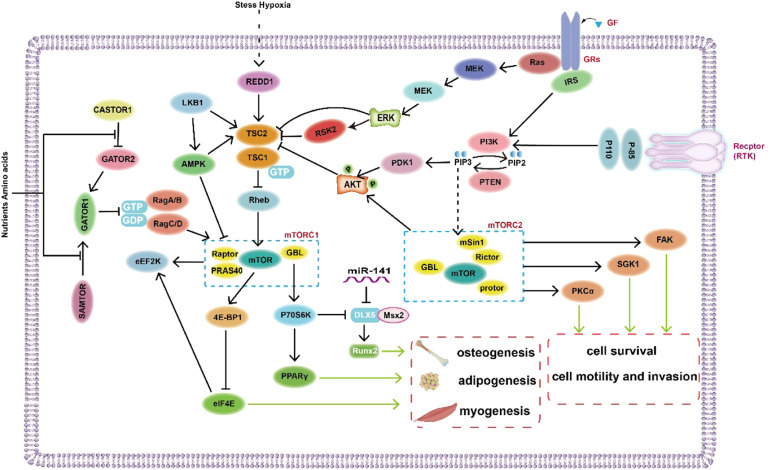
Diagram of the regulatory mechanism of mTOR signaling pathway in mesenchymal stem cells.

AMPK, containing a catalytic subunit (α) and two regulatory subunits (β and γ), is an upstream kinase of mTOR, and the tumor-suppressor protein liver kinase B1 (LKB1) can inhibit the mTORC1 signaling pathway by activating AMPK and TSC2 (Zhao et al., 2019; Sun, 2021) (Figure 2). AMPK activation stimulates pathways that lead to ATP production and that block the synthesis of ATP-consuming factors, such as lipids and cholesterol (Chen et al., 2019; Sun, 2021). In human adipocytes, TNF-α promotes basal glucose uptake and GLUT4 expression through AMPK activation dependent mechanisms (Fernandez-Veledo et al., 2013). Nevertheless, insulin-induced glucose uptake is blocked by AMPK activators, because AMPK stimulation may inhibit glucose transport in insulin-stimulated adipocytes and may inhibit triacylglycerol synthesis to conserve ATP (Fernandez-Veledo et al., 2013). Past studies have shown that once adipocytes differentiate, AMPK activation induces a reduction in the volume of adipocytes by decreasing the activity of enzymes related to triglyceride synthesis, glycerol phosphoryl transferase and acyl CoA (diacylglyceryl transferase) (Habinowski and Witters, 2001). In addition, other studies have suggested that AMPK may have a crucial effect on cell fate determination in human adipose-derived stem cells as a mediator of bone formation and adipogenesis (Fernandez-Veledo et al., 2013). Finally, the Notch signaling pathway has been reported to participate in the regulation of adipogenesis *via* the mTOR signaling pathway (Song et al., 2015).

### 3.2 Mammalian target of rapamycin and osteogenesis

MSCs differentiate into osteoblasts, which are involved in bone formation through the synthesis and sedimentation of mineralized extracellular matrix (Su et al., 2010). Mature osteoblasts eventually become osteoblasts and endoosteocytes or disappear due to apoptosis. Bones are important for mammalian survival, calcium and phosphorus metabolism, and energy homeostasis (Scharla, 2020). Osteoporosis and osteoarthritis are two chronic diseases that are associated with imbalances in bone resorption and formation, and mTOR modulation has been reported to be involved in symptom improvements in certain bone diseases [(Lund-Ricard et al., 2020)]. Researches revealed that mTOR regulates various cellular processes such as growth, proliferation, and differentiation [(Lund-Ricard et al., 2020), (Zhao et al., 2020a)]. In many species ranging from Drosophila to humans, the effect of mTOR signaling on regulatory processes has spurred extensive research (Sun and Liu, 2019). Early studies have suggested that mTOR regulates the function of osteoblasts, and that the mTOR/Raptor/S6K1 signaling pathway is essential for the proliferation and differentiation of osteoblasts [(Chen and Long, 2015), (Dai et al., 2017)]. Although the mTOR signaling pathway may influence osteoblast proliferation and differentiation, earlier studies disputed whether blocking the mTOR signaling pathway with rapamycin affects osteogenesis (Singha et al., 2008).

Bone and dentin are derived from stem cells from apical papilla (SCAPs) that are postnatal MSCs with self-renewing abilities and differentiate into osteoblasts/odontoblasts, adipocytes and nerve cells. According to a recent study, inhibition of the PI3K-Akt-mTOR pathway promotes osteogenic/dentine differentiation in SCAPs *in vitro* and *in vivo* (Tanaka et al., 2018). Recent studies have shown that osteoblasts derived from vascular smooth muscle cells (VSMCs) and MSCs are modulated by autophagy to promote the transformation of calcification signals in vascular structures (Shanahan, 2013; Lee et al., 2014; Caffarelli et al., 2017; Zhou et al., 2021). With the involvement of autophagic proteins, AMPK activation powerfully links Akt/mTOR-associated autophagy to the osteogenic differentiation of MSCs (Zhou et al., 2021). MiR-100-5p and miR-143-3p are involved in regulating the mTOR signaling pathway and promoting osteogenesis (Cen et al., 2021). Moreover, research has shown that decreasing miR-141 in bone tissue alleviates the negative regulation of its target gene Dlx5, indirectly promoting DLX5-Msx2 dimer formation and Runx2 expression (Liu et al., 2016; Cen et al., 2021).

However, there is mounting evidence that the effect of mTOR-mediated autophagy is destructive in bone formation. Rapamycin can block osteoblast proliferation and differentiation in mic and rats (Isomoto et al., 2007; Singha et al., 2008). In contrast, baicalein can stimulate osteoblast differentiation by activating the mTORC1 signaling pathway (Li et al., 2015). PPARγ strongly inhibited Akt/mTOR/p70S6K activity, resulting in osteoblast differentiation and a reduction in the trabecular number (Shen et al., 2016). And epiregulin can promote osteoblast proliferation, and inhibit cell death induced by dexamethasone by activating the Akt/mTOR and Erk/MAPK (mitogen-activated protein kinase) signaling pathways (Fan et al., 2015; Shen et al., 2016).

### 3.3 Mammalian target of rapamycin and chondrogenesis

Limb skeletal elements develop from cartilage templates in a process called chondrogenesis; during chondrogenesis, the aggregated mesenchymal cells undergo a highly ordered process of proliferation and maturation (Shimizu et al., 2007). Chondrogenesis is a key process in bone formation because endochondral ossification requires the formation of cartilage templates (Montero and Hurle, 2007). Osteoblasts and chondrocytes are the most useful cells in osteogenesis and chondrogenesis, respectively (Umezawa and Akutsu, 2008). Chondrogenesis is a rigorously regulated multistep process that includes recruitment/migration of mesenchymal cells, prechondrogenic coagulation of mesenchymal cells, transition to chondrogenic lineage and chondrogenic differentiation (Kang, 2008). There have been many reports on the regulation of the mTOR signaling pathway in chondrogenesis. For example, blebbisatin induces chondrogenesis by activating the PI3K/PDK1/mTOR/p70S6K pathway (Kim et al., 2017b); Akt activity is critical for chondrogenesis but is regulated by mTORC2. Mechanical stimulation combined with low-intensity pulsed ultrasound (LIPUS) promoted TGFβ1-induced chondrogenesis of bone marrow mesenchymal stem cells through the integrin-mTOR signaling pathway (Xia et al., 2017). The PI3K/Akt/mTOR pathway also plays an important role in the regulation of endometrial mesenchymal stromal cells (eMSCs) chondrogenesis, and fluoride can inhibit proliferation and promote autophagy through the PI3K/Akt/mTOR signaling pathway (Ma et al., 2021).

### 3.4 Mammalian target of rapamycin and osteoclastogenesis

Osteoclasts are terminal multinucleated cells that are regulated by nuclear factor-activated T cell C1 (NFATc1) and are responsible for bone absorption (Tong et al., 2020). Enhanced osteoclast formation is an important pathological feature of several age-related bone diseases (Zhang et al., 2005). Osteoclasts participate in bone resorption; bone destruction in rheumatoid arthritis (RA) is caused by osteoclasts and multinucleated cells in the mononuclear/macrophage lineage (Kim and Chen, 2004). Furthermore, bone remodeling is usually a dynamic process regulated by both bone resorbing osteoclasts and bone forming osteoblasts (Wu et al., 2022). Calmodulin-dependent kinase II (CaMKII) regulates osteoclast formation, and the increase in intracellular calcium concentration is the basic process that mediates osteoclast formation (Kim and Chen, 2004; Kang et al., 2020). An imbalance between osteoblasts and bone resorbing osteoclasts is at the heart of many bone diseases (Smink et al., 2009). Exogenous hydrogen sulphide (H_2_S) can promote osteoclast formation by activating the PI3K/AKT/mTOR pathway to down-regulate autophagy (Ma et al., 2020). Similarly, activation of the mTOR signaling is a pivotal player in osteoclast formation induced by *Pasteurella multocida* toxin (PMT) (Kloos et al., 2015). The activation of mTOR in reponse to overloaded orthopedic force can facilitate the osteoblastic differentiation of MSCs (Tian et al., 2021a). In contrast, inhibition of AMPK/mTOR/ULK1 signaling can suppress the formation of osteoclasts by reducing autophagy in glucose-mediated osteoclasts (Cai et al., 2018).

### 3.5 Mammalian target of rapamycin and myogenesis

Myogenesis is a highly regulated multi-step process that refers to the transformation of progenitor cells into multi-nucleated and functional myofibers (Knight and Kothary, 2011). Myogenesis generally occurs in embryonic development or in response to adult muscle damage (Das et al., 2020). In response to adult muscle damage, MSCs differentiate into myoblasts and contribute to muscle tissue homeostasis and regeneration (Shan et al., 2021). Injectable MSCs that at the injured site can differentiate into myogenic cells and further form muscle fibers, are used for the treatment of skeletal muscle injury (Pumberger et al., 2016; Shan et al., 2021). A recent study demonstrated that a ROS-scavenging hydrogel with MSCs facilitates myogenesis to repair injured skeletal muscle, and the gel can enhance MSC proliferation and myogenesis through the PI3K/Akt/mTOR signaling pathway (Shan et al., 2021). Furthermore, mTOR can regulate myogenesis by integrating nutrient availability (Zhang et al., 2016). Abundant evidence has demonstrated that mTOR, as a nutrient sensor, plays a key role in skeletal muscle development (Laplante and Sabatini, 2012). In mice model, deficiency of mTOR alters a series of metabolic statuses of muscles, such as increased basal glucose uptake, impaired redox homeostasis and changed mitochondrial regulation, and then reduces muscle dystrophin content, thereby causing premature death (Risson et al., 2009). Moreover, mTOR can regulate muscle-specific micro-RNAs. For example, mTOR controls miR-1 by affecting the stability of MyoD protein in differentiating myoblasts or regenerating skeletal muscle (Sun et al., 2010). miR-133 and miR-206 are also targets of MyoD and are sensitive to or inhibited by rapamycin during myoblast differentiation (Liu et al., 2007; Williams et al., 2009; Sun et al., 2010). In a rat injury model, the coaction of miR-1, miR-133 and miR-206 mimics can facilitate myogenic differentiation by activating myogenic markers (myoD1, Pax7 and myogenin) (Nakasa et al., 2010). Additionally, mTOR directly affects the expression of miR-17–92 and miR-125b to control skeletal myogenesis (Zhang et al., 2016).

## 4 Effect of mammalian target of rapamycin on the immune response of mesenchymal stem cells

MSCs possess numerous regenerative and immunomodulatory properties (Bottcher et al., 2016). The immunomodulatory properties of MSCs were recognized to play important roles *in vitro* and *in vivo* [(Bartholomew et al., 2002), (Refaie et al., 2021)]. MSCs exert immunoregulatory effects on various immune cells in a cell-cell contact or paracrine manner, which in turn affects the migration, proliferation and differentiation of MSCs [(Chen et al., 2022), (Zhao et al., 2020b)]. MSCs produce many different immunomodulators that regulate the immune function of autologous and allogeneic immune cells, including both innate and acquired immune cells (including T and B cells) (Li et al., 2022). In fact, studies have shown that MSCs affect the metabolic phenotype of activated T cells [(Bottcher et al., 2016), (Pollizzi and Powell, 2014)]. mTOR signaling is a key regulator of glycolysis that increases rapidly to activate normal T cells, and MSC-mediated interference of mTOR signaling is consistent with the T-cell-mediated inhibition of MSCs (Peter et al., 2010). It is now well accepted that targeting mTOR exerts both immunosuppressive and immunostimulatory properties [(Jones and Pearce, 2017), (Weichhart et al., 2015)]. In addition, some MSC-mediated metabolic effects, including the inhibition of mTOR, reduction in glycolysis, and promotion of autophagy, are associated with T-cell-mediated memory formation and longevity [(Kesarwani et al., 2014), (Crompton et al., 2015)].

Studies have shown that the mTOR signaling pathway plays a crucial role in traditional T cells and T-RegS-mediated immune function (Liu et al., 2015). Nitric oxide produced by MSCs inhibits T cells by regulating the LKB1-AMPK-mTOR pathway, thereby inhibiting CD25 translation (Yoo et al., 2017). Moreover, the proinflammatory response mediated by T helper 17 (Th17) cells is increased, while the anti-inflammatory effect mediated by regulatory T (Treg) cells is decreased, exacerbating renal tubular epithelial cell injury (Luo et al., 2021). However, there is considerable evidence that MSCs can control Th17 and Treg cell imbalances (Savio-Silva et al., 2020; Song et al., 2020). By interfering with mTOR signaling, MSCs suppress the differentiation of CD4 (+) T cells into Th17 cells and facilitate Treg cell production (Ghannam et al., 2013; Varco-Merth et al., 2022). Although MSCs suppress normal B-cell proliferation, differentiation, and antibody secretion, CCL2 silencing blocks the suppressive effects on B cells in the MSCs of systemic lupus erythematosus (SLE) patients (Che et al., 2014; Yang et al., 2021a). In a recent study, CCL2 deficiency was shown to enhance synonyms B-cell receptor (BCR) signal transduction through the MST1-mTORC1-STAT1 axis, resulting in a decrease in marginal zone (MZ) B cells and an increase in germinal center (GC) B cells (Yang et al., 2021a). In addition, the suppression of mTORC1 can rescue the aberrant changes in MZ and GC B cells *in vivo* (Yang et al., 2021a).

## 5 The mammalian target of rapamycin signaling pathway in mesenchymal stem cells-mediated treatments of ageing-related diseases

Healthy ageing is a complex biological process characterized by the gradual accumulation of senescent cells and is characterized by stable cell cycle arrest, resulting in impaired homeostasis, impaired regenerative potential, and a gradual decline in the functions of multiple tissues and organs (Shi et al., 2021). Adult stem cells are pivotal for organ-specific regeneration and self-renewal with advancing age, MSCs have become a dependable cell source for stem cell transplantation and are currently being studies in extensive clinical trials (Zhang et al., 2015). The use of MSCs, particularly BMSCs, has therapeutic potential in the treatment of rheumatic diseases and regenerative medicine. In BMSCs, mTOR signaling plays a key role in skewed differentiation and ageing, but its role in inhibiting MSC differentiation remains controversial (Al-Azab et al., 2020). Furthermore, clinical inhibition of this pathway may treat ageing-related diseases, especially osteoporosis and arthritis (Ganguly et al., 2017). Thus, abnormal activation of mTOR signaling plays a crucial role in the treatment of ageing-related diseases by MSCs (Liu et al., 2011; Gharibi et al., 2014).

The aging of MSCs seriously affects their function in stem cell transplantation therapy. In recent years, inhibiting MSC ageing has become the focus of extensive research. Previous reports have suggested that ascorbic acid and coenzyme Q10 suppress MSC senescence, and high glucose induces MSC ageing through Akt/mTOR signaling (Zhang et al., 2015; Zhang et al., 2017; Yang et al., 2018). Moreover, Indian Hedgehog regulates MSC senescence by modulating the ROS/mTOR/4EBP1 and p70S6K1/2 pathways (Al-Azab et al., 2020). However, there are many ageing-related diseases related to MSCs that are also regulated by the mTOR signaling pathway, such as Alzheimer’s disease (AD), Parkinson’s disease (PD), osteoporosis, atherogenesis and so on.

### 5.1 Alzheimer’s disease

AD is a complex, heterogeneous and severe neurodegenerative disease that represents a major form of dementia and is characterized by cognitive behavioral impairment, psychiatric symptoms, progressive cognitive decline, disorientation, behavioral changes and death (Nikolac Perkovic and Pivac, 2019). AD is the most common form of dementia and has huge socio‐economic impacts worldwide (Dong et al., 2019). AD has been historically considered as a disease of gray matter, but accumulating evidence has suggested that white matter alteration occurs in the disease course (Sachdev et al., 2013). MSCs have received much attention for their potential in regenerative medicine, and they offer new hope as a therapeutic strategy for neurodegenerative disease treatment, including AD (Divya et al., 2012; Farahzadi et al., 2020). New studies have shown that multiple signaling pathways are involved in the pathophysiology of AD, the most important of which include mTOR, AMPK, glycogen synthase kinase 3 (GSK3), and Wnt3/β-catenin (Godoy et al., 2014; Tramutola et al., 2015). Therefore, the effect of MSCs on nerve cells as an AD treatment may be largely related to the mTOR signal transduction network. In addition, autophagy has been shown to reduce the proliferation of BMSCs treated with amyloid-β_1-42_ (Aβ_1-42_) through the Akt/mTOR signaling pathway (Yang et al., 2019).

### 5.2 Parkinson’s disease

PD is an ageing-related neurodegenerative disorder characterized by the loss of dopaminergic neurons in the substantia nigra midbrain region and the presence of intracytoplasmic inclusions called Lewy bodies (Dexter and Jenner, 2013; Anderson and Maes, 2014; Safari et al., 2016). When nearly 60% of dopaminergic neurons in the aubstantia nigra dense region are eliminated, Parkinsonian symptoms begin to appear (Cooper et al., 2009). The occurrence of PD is related to many factors, such as ageing and oxidative stress (Ma et al., 2022). It is estimated that approximately 12% of people 65 and older have PD (Lan et al., 2017). In advanced PD patients, the glutathione level is lower than that in the age‐matched control group, which suggests that the disease course may be involved in the vulnerability of the substantia nigra pars compacta (SNc) to oxidative stress (Salaramoli et al., 2022). In previous studies, the combination of granulocyte colony-stimulating factor and BMSCs has beneficial effects on a PD model (Ghahari et al., 2020). There have been few reports of the direct involvement of MSCs and regulation of the mTOR signaling pathway in PD, but increasing evidence indicates that mTOR plays key roles in the pathogenesis of PD (Zhu et al., 2019). The mTOR protein, which is a key regulator of cell metabolism and survival, has emerged as a novel therapeutic target for PD.

### 5.3 Osteoporosis

Osteoporosis is a major risk cause of fracture or broken bones in later life (Otonari et al., 2021). In older individuals, the rate of bone resorption exceeds the rate of bone formation, resulting in bone loss (Owen-Woods and Kusumbe, 2022). Osteoporosis is a major public health problem and a heterogeneous disease with significant socioeconomic importance because it is associated with fracture and appropriate early intervention can greatly alleviate the problem before fracture occurs (Pietschmann and Peterlik, 1999; Jordan and Cooper, 2002). The decline in MSC proliferation and stem cell properties with age is also thought to account for the gradual decrease in bone mass and reduced risk of osteoporosis and fracture in older adults (Bellantuono et al., 2009).

BMSCs isolated from elderly individuals or aged animals exhibit decreased bone marrow frequencies and proliferation rates and higher levels of ageing and ageing-related changes than young cells (Gharibi et al., 2014). It has been reported that activation/inhibition of mTOR signaling positively/negatively regulates BMSC/osteoblast-mediated bone formation, adipogenic differentiation, osteoblast homeostasis, and osteoclast mediated bone resorption, leading to altered bone homeostasis, which can lead to or prevent osteoporosis (Shen et al., 2018). Other studies have shown that amyloid β induces osteoporotic defects *in vivo* and *in vitro* through mTOR and autophagy, and the regulation of amyloid β on BMSCs is dependent on mTOR, thus providing a possible mechanism for osteoporotic remodeling in AD patients (Yang et al., 2019; Lin et al., 2021).

### 5.4 Atherosclerosis

Atherosclerosis is a common age-related disease, and increasing age is one of the major risk factors for developing atherosclerosis (Wang and Bennett, 2012). One of the phenotypic manifestations of the arteries is atherosclerosis, which is a chronic inflammatory disease (Poznyak et al., 2022). Primary atherosclerosis may be a phenotypic feature of mitochondrial disease. It has been suggested that atherosclerosis may be a major manifestation of metabolic defects (Finsterer, 2020). In many cases, atherosclerosis is the root cause of vascular disease, including heart disease and stroke (Kazemi-Bajestani and Ghayour-Mobarhan, 2013). Previous studies have shown that mesenchymal stem cells (MSCs) have therapeutic effects on a variety of diseases, including atherosclerosis (Yang et al., 2021b). Maldifferentiation of mesenchymal stem cells and maladjustment of cell fate programs associated with age and metabolic diseases may exacerbate arteriosclerosis due to excessive transformation into osteoblast-like calcified cells (Schaub et al., 2019). As a chronic vascular inflammatory disease, atherosclerosis has been demonstrated to exert immunomodulatory and immunosuppressive effects on MSCs by secreting humoral factors (Takafuji et al., 2019). It has been reported that Sestrin2 is a stress-inducing protein that inhibits mTOR by activating AMPK (Tian et al., 2021b). Furthermore, Sestrin2 is strongly associated with atherosclerosis, suggesting that inhibition of mTOR signaling can be used to treat atherosclerosis (Tian et al., 2021b). In other words, mTOR is a target for the treatment of atherosclerosis.

### 5.5 Other diseases

The mTOR signaling pathway is involved in the development of many human diseases, and its dysregulation has been reported in several pathological processes, especially in age-related human diseases and in mouse models of accelerated aging (Lee et al., 2020). In addition to the specific diseases mentioned above, mTOR signaling is also closely related to the occurrence and development of many major diseases related to aging, such as cardiovascular diseases and bone disease (He and Liu, 2014). In the cardiovascular system, the mTOR signaling can regulate angiogenesis (Samidurai et al., 2018; Liu et al., 2022). Angiogenesis depends on the full function of vascular smooth muscle cell progenitors such as pericytes and their circulating counterparts mesenchymal stromal cells (Schaub et al., 2019). MSCs are located around blood vessels, and hematopoietic stem cells (HSCs) and MSCs occupy special microenvironments (Owen-Woods and Kusumbe, 2022). Blood vessel wear is a precursor to aging, and loss of vascular density and pericytes is a sign of aging of organs and tissues (Chen et al., 2021a). Therefore, vascular vessels and microenvironments provide secretory signals to affect cell function in age-related diseases, except regulating organ development and stem cell behavior (Chen et al., 2021b; Owen-Woods and Kusumbe, 2022).

Moreover, the Notch pathway is one of several pro-proliferative pathways activated by mTOR (Ivanovska et al., 2017). Notch signaling which negatively controls angiogenesis, is an important component of crosstalk between vascular cells and bone lineage cells in the process of bone formation and remodeling (Chen et al., 2020). Activation of the VEGF-Notch signaling pathway may restore the proliferation of MSCs in patients with aplastic anemia (Deng et al., 2019). Anyhow, crosstalk between the Notch and mTOR pathways may play important roles in MSC-mediated senile diseases. In addition, bone mesenchymal cells also undergo age-dependent alterations (Chen et al., 2020). In osteoarthritis patients, TGF-β1 and mTOR are highly expressed, and blocking TGF-β signaling in the MSCs of subchondral bone deadens osteoarthritis (Zhen et al., 2013; Owen-Woods and Kusumbe, 2022). In mice model, osteoarthritis was induced by TGF-β1 overexpression, which resulted in increased mTOR expression (Davidson et al., 2007; Wen et al., 2021; Owen-Woods and Kusumbe, 2022). In addition, previous studies have revealed that PI3K/AKT/mTOR signaling is involved in the important regulation of ischemic brain injury and tumors (Xu et al., 2020).

## 6 Discussion and conclusion

We summarized relevant studies on the involvement of mTOR in the differentiation of adipocytes, bone and muscle. We found that the mTOR signaling pathway in MSCs is crucial. Inhibition or activation of this pathway can affect cell differentiation of MSCs. In addition, there are many mTOR related signaling pathways that have a certain impact on the immune response, and regulate angiogenesis, such as the Notch pathway. Therefore, in the future, the influences caused by mTOR may be regulated by other pathways. What are the difficulties in identifying these new approaches? What are the disadvantages of these new pathways compared to the mTOR pathway? Is the activation time of other regulatory pathways different from the time or stage of activation of the mTOR signaling pathway? Is it cost-effective to regulate MSC differentiation through these new pathways? These questions are all worth considering.

In recent years, there has been a sharp increase in the number of articles on the activation and inhibition of the mTOR signaling pathway, and many researchers have focused on the development of innovative inhibitors. Most of the reported inhibitors target mTORC1 or PI3K and mTOR dual inhibitors, including competitive inhibitors and allosteric inhibitors. The discovery of rapamycin-insensitive mTORC2 stimulated the development of mTOR inhibitors that target the kinase domain (el Hage and Dormond, 2021). However, we need to identify more effective activators that target positively regulated pathways. Therefore, we can attempt to develop new inhibitors that negatively regulate pathways or activators that positively regulate pathways by exploring the relevant mechanisms of mTORC2. Perhaps we can find more effective inhibitors or activators by examining other upstream or downstream factors in the mTOR signaling pathway. Moreover, although senile diseases are a difficult problem facing society, there have been few studies on MSCs or the regulation of the mTOR signaling pathway. In particular, research on PD is scarce, but it is a typical disease worthy of further study. Therefore, there is still considerable space and prospects in this field, which is worthy of further exploration. We believe that these problems will be solved in the future, so the mTOR signaling pathway is still worthy of investigation.
